# Genomic and transcriptomic insights into the virulence and adaptation of shock syndrome-causing Streptococcus anginosus

**DOI:** 10.1099/mic.0.001535

**Published:** 2025-02-20

**Authors:** Yu-Juan Lin, Chih-Ho Chen, Ian Yi-Feng Chang, Ruei-Lin Chiang, Hsing-Yi Wang, Cheng-Hsun Chiu, Yi-Ywan M. Chen

**Affiliations:** 1 Department of Microbiology and Immunology, College of Medicine, Chang Gung University, Taoyuan, Taiwan, ROC; 2 Department of Pediatrics, Kaohsiung Chang Gung Memorial Hospital, Kaohsiung, Taiwan, ROC; 3 Molecular Medicine Research Center, Chang Gung University, Taoyuan, Taiwan, ROC; 4 Department of Neurosurgery, Chang Gung Memorial Hospital at Linkou, Taoyuan, Taiwan, ROC; 5 Genomic Medicine Core Laboratory, Chang Gung Memorial Hospital, Taoyuan, Taiwan, ROC; 6 Molecular Infectious Disease Research Center, Chang Gung Memorial Hospital, Linkou, Taiwan, ROC; 7 Graduate Institute of Biomedical Sciences, College of Medicine, Chang Gung University, Taoyuan, Taiwan, ROC

**Keywords:** genome, *Streptococcus anginosus*, transcriptome

## Abstract

*Streptococcus anginosus* is a common isolate of the oral cavity and an opportunistic pathogen for systemic infections. Although the pyogenic infections caused by *S. anginosus* are similar to those caused by *Streptococcus pyogenes*, *S. anginosus* lacks most of the well-characterized virulence factors of *S. pyogenes*. To investigate the pathogenicity of *S. anginosus*, we analysed the genome of a newly identified *S. anginosus* strain, KH1, which was associated with toxic shock-like syndrome in an immunocompetent adolescent. The genome of KH1 contains nine genomic islands, two Clustered Regularly Interspaced Short Palindromic Repeats (CRISPR)/CRISPR-associated systems and many phage-related proteins, indicating that the genome is influenced by prophages and horizontal gene transfer. Comparative genome analysis of 355 *S*. *anginosus* strains revealed a significant difference between the sizes of the pan genome and core genome, reflecting notable strain variations. We further analysed the transcriptomes of KH1 under conditions mimicking either the oral cavity or the bloodstream. We found that in an artificial saliva medium, the expression of a putative quorum quenching system and pyruvate oxidase for H_2_O_2_ production was upregulated, which could optimize the competitiveness of *S. anginosus* in the oral ecosystem. Conversely, in a modified serum medium, purine and glucan biosynthesis, competence and bacteriocin production were significantly upregulated, likely facilitating the survival of KH1 in the bloodstream. These findings indicate that *S. anginosus* can utilize diverse mechanisms to adapt to different environmental niches and establish infection, despite its lack of toxin production.

**Impact Statement**

Microbes of the *Streptococcus anginosus* group (SAG) have been recognized as pathobionts capable of causing various invasive infections. Nevertheless, little is known regarding the pathogenicity of these bacteria. SAG bacteria exhibit considerable genetic diversity between strains, but how the diversity contributes to the transition from commensal to pathogen is unclear. Here, we analysed the genome and transcriptomes of a shock syndrome-causing *S. anginosu*s strain, KH1, in growth conditions mimicking the oral cavity and the bloodstream to investigate key factors for a successful transition from commensal to pathogen. Genomic analysis of KH1 is consistent with previous findings that the genome sequence is related to the origin of the isolate. Transcriptomic analysis indicates that the ability to adapt to different environments, rather than toxin production, is a key mechanism by which *S. anginosus* can cause invasive infection.

## Data Availability

The data supporting the findings are available within the article or supplementary files. The raw RNA-seq reads have been deposited in the NCBI GEO database under the assigned accession number GSE285825. The accession number of the *Streptococcus anginosus* KH1 genome is CP117042.1. The accession number of the raw reads of KH1 transcriptomes is GSE285825.

## Introduction

*Streptococcus anginosus*, a member of the *S. anginosus* group (SAG) [1], is commonly found in the mucosal membranes of the oral cavity [2]. All species of SAG, i.e. *S. anginosus*, *Streptococcus constellatus* and *Streptococcus intermedius*, can spread from the oral cavity to the upper respiratory, digestive and reproductive tracts and are considered part of the normal microbiome. However, SAG species have been frequently implicated in pyogenic infections [3], which are characterized by acute inflammatory responses and pus formation. For instance, a population-based surveillance study in Canada revealed that the incidence of SAG infections was higher than those caused by *Streptococcus pyogenes* and *Streptococcus agalactiae* [4]. More recent research further confirmed that bacteremia caused by SAG could result in significant morbidity and mortality [5]. The suppurative infections caused by SAG resemble those caused by *S. pyogenes*, including pharyngitis, bacteremia and serious deep tissue infections [4,6,8], indicating that the pathogenic potential of SAG may be greater than previously expected. Although not all SAG infections result in pyogenic outcomes [9], recent case reports and reviews also suggest that SAG species are common pathogens in conditions such as descending necrotizing mediastinitis [10], intracranial infections [11,12] and even osteomyelitis [13]. Furthermore, studies have shown that *S. anginosus* could co-exist with cystic fibrosis (CF)-associated *Pseudomonas aeruginosa* DWW2 in biofilms and enhance *P. aeruginosa* DWW2 pyocyanin production*,* resulting in increased virulence in the infection mode [14,15]. Although the role of *S. anginosus* in CF pathogenesis requires further investigation, this study suggests that *S. anginosus* could modulate the virulence of cohabitating microbes. Lastly, a recent study identified an enrichment of *S. anginosus* in the gastric microbiome of patients with gastric cancer, and the enrichment is inversely correlated with the presence of *Helicobacter pylori* [16]. The association between *S. anginosus* and gastric tumorigenesis was further investigated in a mouse model, confirming the pathogenic potential of *S. anginosus* [17].

While the clinical significance of SAG is evident, the virulence mechanisms of SAG remain poorly understood [2]. To date, the few studied virulence factors of *S. anginosus* include a laminin-binding protein that mediates the adhesion of *S. anginosus* to the exposed basement membrane of heart valve tissues during the course of endocarditis [18], a fibronectin-binding protein that enhances the interaction of *S. anginosus* F4 with polymorphonuclear leukocytes [19], and homologues of *S. pyogenes* streptolysin S, a haemolysin produced through the *sag* operon expression [20,21]. However, not all *S. anginosus* strains possess the *sag* cluster. To be noted, genes encoding the streptococcal pyrogenic exotoxins, which are implicated in invasive infections, are absent in * S. anginosus*. Though, studies have shown that the LuxS-based, autoinducer-II signalling pathway regulates biofilm formation in *S. anginosus* [22] and that *S. anginosus* exhibits antibiotic resistance [23], suggesting that, similar to other streptococcal species, virulence expression is regulated by environmental growth conditions in *S. anginosus*.

Genomic studies have revealed that the CRISPR-Cas systems, prophages and mobile genetic elements are present among * S. anginosus* strains [24,26]. Furthermore, *S. anginosus* exhibits natural competence and, thus, can acquire virulence genes, such as antibiotic resistance genes, through horizontal gene transfer (HGT). For instance, it has been hypothesized that *S. anginosus* strain 47S1 acquires genes encoding resistance to tetracycline, aminoglycosides, fusidic acid and fluoroquinolone via HGT [24]. The presence of *vanG*, encoding vancomycin resistance, has also been described in *S. anginosus* SA1 [27]. In addition, a comparative genomic study of *S. anginosus* JA4206 revealed unique genes and variants that could contribute to the acquisition of daptomycin resistance [26].

A strain of *S. anginosus*, designated KH1, was recently isolated from a previously healthy, young patient at Kaohsiung Chang Gung Memorial Hospital [28]. This patient presented with streptococcal toxic shock-like syndrome 2 days following a dental procedure for caries, suggesting that KH1 likely originated in the oral cavity. Although a narrow haemolytic zone developed around the colony on a blood agar plate [28], the *sag* genes were not identified by PCR. To investigate the activity that enables KH1 to establish systemic infections, we determined the genome and analysed the transcriptomes of KH1 cultivated in environments mimicking the oral cavity and the bloodstream. The genomic analysis identified nine genomic islands, reflecting its natural competence. We also found that the transcriptomes were significantly regulated by growth media, indicating that *S. anginosus* could modify its transcriptome to facilitate survival in specific niches.

## Methods

### Bacterial strains and growth conditions

*S. anginosus* KH1 was routinely cultivated in Todd Hewitt (TH, Difco) broth, at 37 °C in a 10% CO_2_ atmosphere. For transcriptomic analysis, KH1 was cultivated in either artificial saliva medium (SV) or modified serum medium (SM). The artificial SV was prepared as per Wong and Sissons [29], with some modifications, and contains 0.25% mucin, 1% proteose peptone, 1% trypticase peptone, 1% yeast extract, 0.25% KCl and 0.25% glucose. To prepare the modified SM, venous blood samples were collected from healthy human volunteers in BD Vacutainer™ SST™ II Advance Tubes (BD). The blood samples were subjected to centrifugation at 2500 ***g***, 25 °C for 10 min to remove the blood cells. The recovered plasma was heated at 56 °C for 30 min to inactivate complement. HEPES and glucose were added to the plasma to final concentrations of 40 mM and 0.5%, respectively, to make modified SM.

To determine growth kinetics, overnight cultures of KH1 were grown in TH broth and subcultured into pre-warmed artificial SV or modified SM at 1 : 10, 1 : 20 and 1 : 40 dilutions. The growth at OD_600_ was monitored using a Bioscreen C Microbiology Reader (Oy Growth Curve AB Ltd.). For each condition, three biological replicates were analysed.

### Purification of chromosomal DNA, sequencing and annotation

Total cellular DNA was isolated from a mid-exponential (OD_600_=0.6) phase culture of *S. anginosus* KH1 as described previously [30]. Genome sequencing was performed by a commercial service (Genomics, Taiwan). Briefly, the genomic DNA was sheared using g-TUBE (Covaris), and the library was prepared using the SMRTbell express template Prep Kit 2.0 (PacBio). The PacBio Sequel/Sequel II platform was used to determine the genome sequence. A primary filter analysis was performed on the Sequel instrument to trim the low-quality regions, and the secondary analysis was performed using the SMRT analysis pipeline version 8.0 (https://www.pacb.com/support/software-downloads/). The reads were assembled initially using Hifiasm v0.8 with the default setting [31]. The contigs were further assembled using SSPACE-Long v1.1 [32]. ORFs were predicted and annotated using Prokka v1.12 (https://github.com/tseemann/prokka). Clusters of Orthologous Groups (COGs) were analysed using eggNog-mapper 2.1.12 [33], and the Kyoto Encyclopedia of Genes and Genomes (KEGG) pathway was used to predict gene functions. Genomic islands and CRISPR systems were identified through IslandViewer 4 [34] and CRISPRCasFinder 4.2.20 [35], respectively. Putative antibiotic resistance loci were analysed by Comprehensive Antibiotic Resistance Database (CARD) [36]. The complete genome sequence of *S. anginosus* KH1 has been deposited in the GenBank database with the accession number CP117042.1.

### RNA isolation and transcriptomic analysis

Total cellular RNA was isolated from KH1 cultures at OD_600_=0.3 as described [37] and further purified using the RNeasy kit (Qiagen). rRNA was removed from the RNA preparation using RiboMinus transcriptome isolation kits (Invitrogen). PCR was performed with primers specific for PO908_05840 to ensure that the preparations were free of chromosomal DNA. Transcriptome sequencing was performed by a commercial service (Welgene, Taiwan). Briefly, the RNA library was constructed using a SureSelect XT HS2 mRNA library preparation kit (Agilent, USA). The sequences were determined using Illumina’s sequencing-by-synthesis technology (Illumina, USA). Reads were assessed through a Phred quality score (Q score of 20) and trimmed using Trimmomatic v0.36 [38]. The trimmed reads were mapped to the genome sequence of KH1 by using HISAT2 [39]. TPM (transcripts per million) was used to calculate the expression level of each gene. The significant difference in the expression level of each locus between the two growth conditions was analysed using DESeq2 v1.28.1 [40] with genome bias detection/correction and Welgene Biotech’s in-house pipeline. For each condition, duplicate samples were analysed. Genes exhibiting a fold change >2.0 and a *q*-value <0.05 between the two culture conditions were considered significant. The raw RNA-seq reads have been deposited in the NCBI GEO database under the accession number GSE285825.

### Comparative genomic analysis

We used panaroo (v1.5.0) [41] to generate the pan-genome of 355 *S*. *anginosus* strains (Table S1, available in the online Supplementary Material). Among these genomes, gene annotation files of 275 genomes were downloaded from the NCBI database (accessed on 19 July 2024). For 79 incomplete genomes without gene annotation files (accessed on 19 July 2024), we predicted and annotated the incomplete genome by Prokka [42]. The phylogenetic tree of these strains and KH1 was generated using iqtree2 (v2.1.2) [43]. The core genome contains orthologues from all strains with ≥99% identity.

### Construction of recombinant *S. anginosus* strains

Recombinant *S. anginosus* strains deficient in *yodI*, *alr* and *lacB* were generated by ligation mutagenesis [44]. In short, two DNA fragments flanking the target region were amplified from *S. anginosus* KH1 by PCR using specific primers. The resulting products were restriction-digested and mixed with DNA fragments containing a nonpolar erythromycin (Em) resistance gene (*erm*) [45] in a ligation reaction. The ligation mixture was introduced into KH1 by natural transformation. The allelic exchange event in the Em-resistant transformants was verified by colony PCR with primers located outside the insertion site of the *erm* gene. All primers used in mutant construction are listed in Table S2.

### Determination of MIC and minimal bactericidal concentration

The MIC for vancomycin in *S. anginosus* strains was determined using a 96-well plate assay, following Clinical and Laboratory Standards Institute guidelines. The plates were incubated at 37 °C under 10% CO_₂_ for 24 h. The minimal bactericidal concentration (MBC) was subsequently assessed by subculturing samples from wells without visible growth onto TH agar plates to determine the lowest bactericidal concentration. For each strain, three biological replicates were analysed.

## Results and discussion

### The general features of the *S. anginosus* KH1 genome

**Fig. 1.**
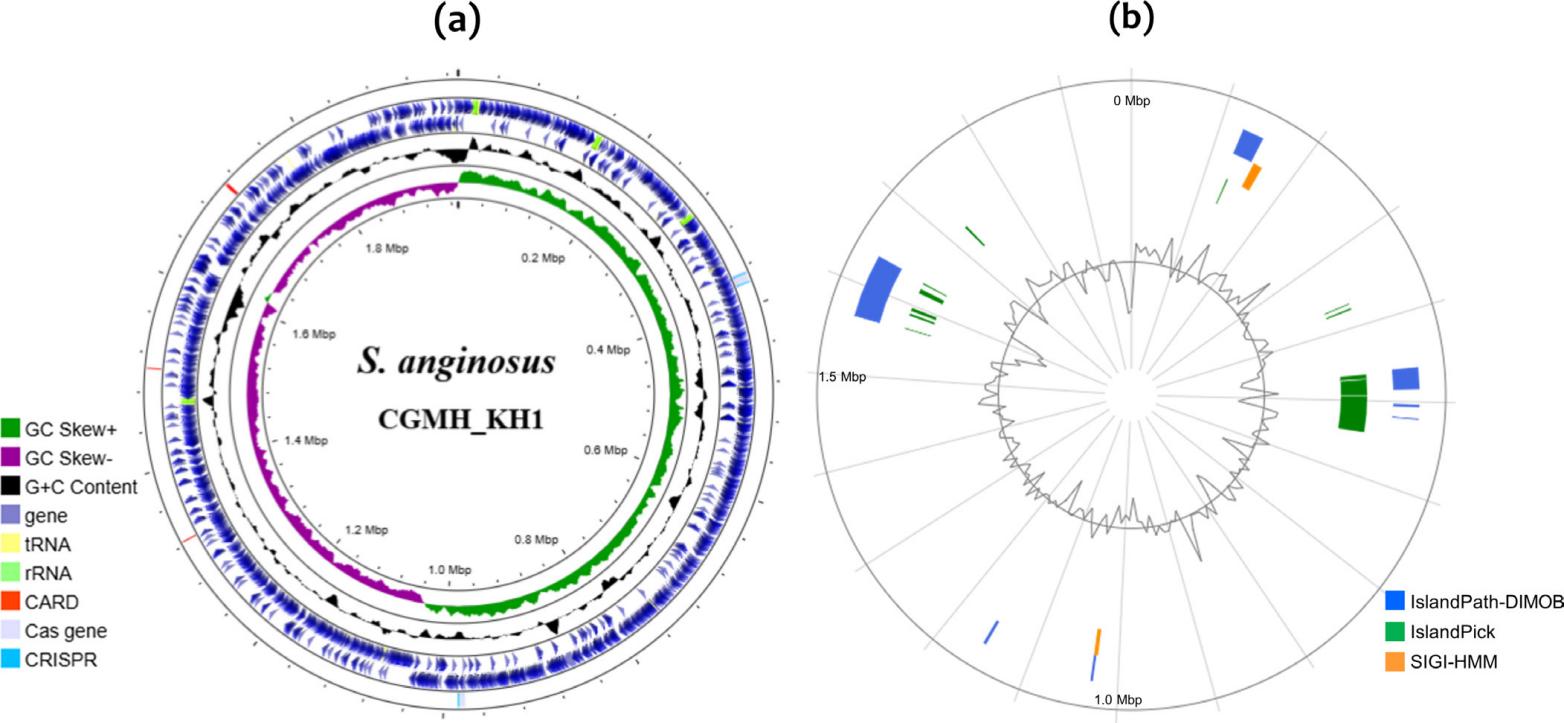
Genome structure of *S. anginosus* KH1. (**a**) Circular representation of the chromosome. The genome position, scaled in Mbp, from base 1 is shown on the inner circle. From inner to outer circles, the second circle shows the GC skew curve (10 kbp window and 0.1 kbp incremental shift). The values for plus and minus strands are shown in green and purple, respectively. The third circle shows the G+C content (in 10 kb window, black). The values that are greater than, and below, the average (38.81%) are shown. The fourth circle shows the CDs. Genes are in light purple. tRNA and rRNA are in yellow and mint, respectively. The outermost circle shows loci predicted by CARD (red), the predicted *cas* genes (light lavender) and CRISPRs (sky blue). (**b**) Circular representation of the predicated genomic islands. The inner circle displays the G+C content. The middle circles indicate loci predicted by IslandPath-DIMOB (cobalt blue), IslandPick (green) and SIGI-HMM (orange).

The general features of the KH1 genome are listed in Fig. 1. Briefly, KH1 harbours a circular chromosome of 1 970 657 bp, with a G+C content of 38.81 mol%. The complete genome contains 2026 predicted CDs, including 12 rRNA in four operons, 61 tRNA and 3 ncRNA. Two CRISPR-Cas systems were identified in the genome by CRISPRCasFinder [35]. Based on blast analysis, one belongs to type II-A of the class II CRISPR-Cas systems, and the other belongs to type III-A of the class I systems. While type II-A is commonly found in *S. anginosus* [46], both types II-A and III-A are dominant subtypes in streptococci [47]. In line with other *S. anginosus* genomes, putative phage-related genes were annotated in the genome. The presence of CRISPR-Cas systems and phage proteins suggests that the genome of KH1 is influenced by HGT.

Similar to other *S. anginosus* genomes, loci involved in metabolism (COG categories C, E, F, G, H, I, P and Q) and information processing and storage (COG categories J, K and L) are the most abundant categories, comprising 31.7 and 24.6% of the genome, respectively (Table S3). This genome also harbours more than 500 poorly characterized loci, covering 28.4% of the genome. A relatively high percentage (10.35%) of hypothetical proteins was observed in the KH1 genome, compared to a subset of 13 complete *S. anginosus* genomes (7.2–12.0%), which included strains that are phylogenetically distant from or relatively close to KH1 (see below for a detailed description of the phylogenetic analysis). The proportion of hypothetical proteins in *S. anginosus* KH1 is notably higher than that in *S. pyogenes* A20 (CP003901, 8.8%), isolated from a patient with necrotizing fasciitis [48]. Interestingly, the KH1 genome features a duplicated region of about 39 kbp. The duplicated genes exhibit point mutations, resulting in ORF variations. One region contains the PO908_02445–02760 CDs, while the other contains the PO908_02765–03075 CDs. To be noted, the 39 kbp duplicated region also contains a genomic island coding for 14 genes; thus, the KH1 genome contains two identical islands, designated island 2 (PO908_02570–02635) and island 4 (PO908_02895–02960). The genomic island in the duplicated region is described further below.

### Putative antibiotic resistance loci

**Fig. 2.**
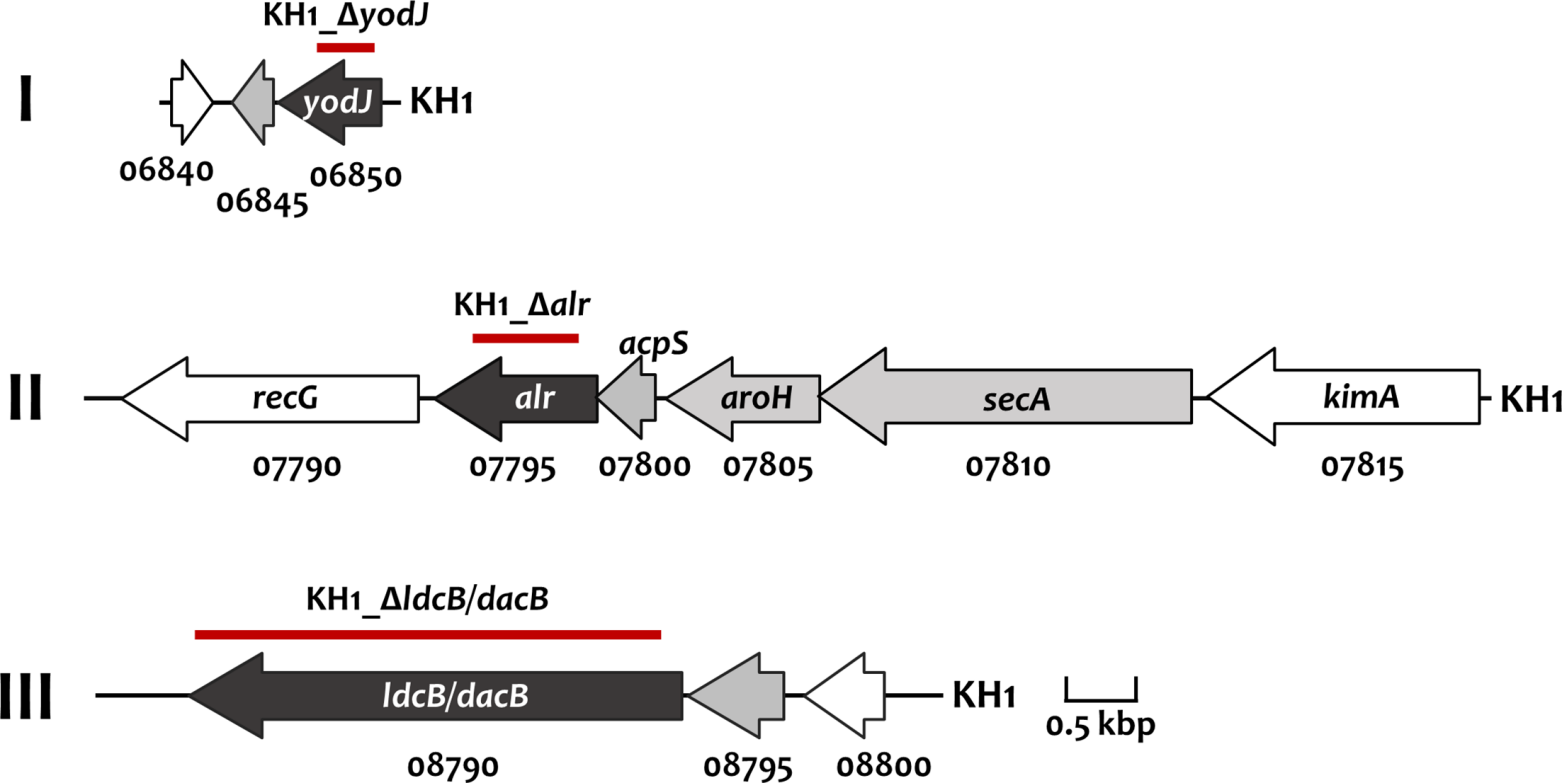
Schematic representation of the putative antibiotic resistance loci in *S. anginosus* KH1. The relative location and orientation of each ORF are shown. The proposed gene name based on blastp analysis is indicated within or above the gene. The PO908_tag number is listed below the gene. Genes identified by CARD are in black, and loci predicated to be in the same operon are in grey. The regions replaced by non-polar *erm* in the mutant strains are indicated by a thick red line and listed above the gene cluster.

Three putative vancomycin resistance loci, *yodJ*, *alr* and *ldcB/dacB*, were predicted in the KH1 genome by CARD [36] (Table 1). Based on the distance between the flanking genes, it is predicted that both *yodJ* and *ldcB/dacB* are part of two-gene operons (operons I and III), and *alr* is the last gene of a four-gene operon (operon II, Fig. 2). The predicted two-gene operon containing *yodJ* and PO908_06845 (operon I) encodes a putative carboxypeptidase and an AraC family ligand binding domain-containing protein, respectively. PO908_06845 does not possess a helix-turn-helix DNA binding domain, and thus, its function in the expression regulation of operon I is unknown. Transcriptomic analysis of operon I demonstrated low expression levels for its constituent genes in either the artificial SV or the modified SM (Table 1).

**Table 1.** Expression of the putative antibiotic resistance genes of *S. anginosus* KH1

Locus tag*	Gene†	ARO‡	Description	TPM	
				SM§	SV§
**Operon I**					
PO908_06845	–	–	AraC family ligand binding domain-containing protein	15.0	15.8
PO908_06850	*yodJ*	*vanY* of *vanG* cluster	Putative carboxypeptidase YodJ	41.3	33.6
**Operon II**					
PO908_07795	*alr*	*vanT* of *vanG* cluster	Alanine racemase	506.1	152.1
PO908_07800	*acpS*	–	Holo-[acyl-carrier-protein] synthase	345.1	113
PO908_07805	*aroH*	–	Phospho-2-dehydro-3-deoxyheptonate aldolase	349.4	156.7
PO908_07810	*secA*	–	Translocase subunit SecA	674.4	444.5
**Operon III**					
PO908_08790	*ldcB/dacB*	*vanY* of *vanB* cluster	ld-Carboxypeptidase LdcB/DacB	307.8	102.6
PO908_08795	–	–	DUF6287 domain-containing protein	91	49.2

*Loci identified by CARD are shaded in Fig. 2. All three operons are transcribed from the minus strand.

†Proposed gene name.

‡Best-hit Antibiotic Resistance Ontology predicted by CARD.

§SM, modified SM; SV, artificial SV.

Operon II consisting of four genes, *secA-aroH-acpS-alr*, encodes translocase SecA, an aldolase, holo-[acyl-carrier-protein] synthase and an alanine racemase, respectively. Expression levels of the operon II genes were elevated by 1.5–3-fold when the organism was grown in modified SM, compared to expression derived from growth in artificial SV.

Lastly, operon III, PO908_08795-*ldcB/dacB*, encodes a hypothetical protein and a putative ld-carboxypeptidase, respectively. The putative LdcB/DacB contains a YSIRK-type signal peptide at the *N*-terminus, three bacterial SH3 domains and four group B *Streptococcus* protein-like domains in the central part, followed by a conserved carboxypeptidase domain of the LdcB superfamily. Similar to operon II, the expression of this operon was also upregulated when the organism was grown in the modified SM, compared to the artificial SV.

An attempt was made to investigate whether these genes are involved in vancomycin resistance using isogenic mutant strains. *S. anginosus* KH1 is very sensitive to vancomycin, with a MIC at 1 μg ml^−1^ and a MBC at 64 μg ml^−1^. Inactivation of *yodJ*, *alr* or *ldcB/dacB* did not increase the sensitivity of *S. anginosus* KH1 to vancomycin significantly. All mutant strains exhibited the same MIC and MBC as WT KH1. Thus, the functions of these genes in antibiotic resistance are unclear.

### Genomic islands

The genomic islands were predicted using IslandViewer 4 (Fig. 1b), which incorporates IslandPath-DIMOB [49], IslandPick [34] and SIGI-HMM [50]. Regions identified by at least two algorithms were recognized as an island. This process resulted in the discovery of nine genomic islands (Tables 2 and S4–S11). All islands exhibited a G+C content lower than the overall genome (38.81%), thereby confirming their origin by HGT. Although many proteins encoded by genes on the islands are functionally unknown, most of the islands harbour loci encoding phage proteins, proteins related to conjugal transfer or a toxin–antitoxin system. The expression of most genes on islands 2, 3 and 4 (Tables S5 and S6) was upregulated in KH1 cultures grown in the modified SM; however, as most of the loci were of unknown function, the role of these proteins for growth in the bloodstream remains unknown. It was also observed that most of the genes on islands 1 and 5 (Tables S4 and S7), which include phage proteins (PO908_00730 and 00765 of island 1) and a putative TonB-dependent receptor (PO908_05375 of island 5), exhibited enhanced expression in the artificial SV, compared to expression in the modified SM, suggesting that these genes may play a role in adaptation to the oral cavity environment. The impact of culture medium on the expression of loci located on islands 6, 7, 8 and 9 was less straightforward (Tables S8–S11). To be noted, expression of loci encoding proteins related to the toxin–antitoxin system, i.e. PO908_08055 of island 6 and PO908_08330, 08365 and 08435 of island 9, did not appear to be altered by the bacterial growth medium, suggesting that these proteins may be required in both the oral cavity and in the bloodstream.

**Table 2.** Properties of the genomic islands in *S. anginosus* KH1

Island	Position (nt)*	Locus tag	Total genes	%G+C content	Proposed functions	Identified by†
1	129 037–133 832	PO908_00725–00775	11	38.14	Remnants of prophages	1, 2
2‡	477 301–484 453	PO908_02570–02635	14	34.41	Remnants of prophages	1, 2
3	501 193–513 503	PO908_02750–02860	23	36.53	Remnants of prophages	1, 2
4‡	516 247–523 399	PO908_02895–02960	14	34.41	Remnants of prophages	1, 2
5	1 025 622–1 030 166	PO908_05375–05400	6	32.83	Unknown	1, 3
6	1 565 359–1 570 898	PO908_08050–08085	8	38.23	Toxin–antitoxin system	1, 2
7	1 584 282–1 598 180	PO908_08170–08220	11	33.22	Conjugal transfer	1, 2
8	1 612 764–1 615 030	PO908_08300–08310	3	30.83	Unknown	1, 3
9§	1 615 732–1 635 653	PO908_08320–08445	26	37.92	Prophage-related proteins, toxin–antitoxin system and conjugal transfer	1, 2

*The location of the island on KH1 genome.

†1, IslandPath-DIMOB; 2, IslandPick; 3, SIGI-HMM.

‡Islands 2 and 4 are identical.

§PO908_08390–08405 of island 9 were predicted only by IslandPath-DIMOB.

### Pan genome construction and phylogenetic analysis

**Fig. 3.**
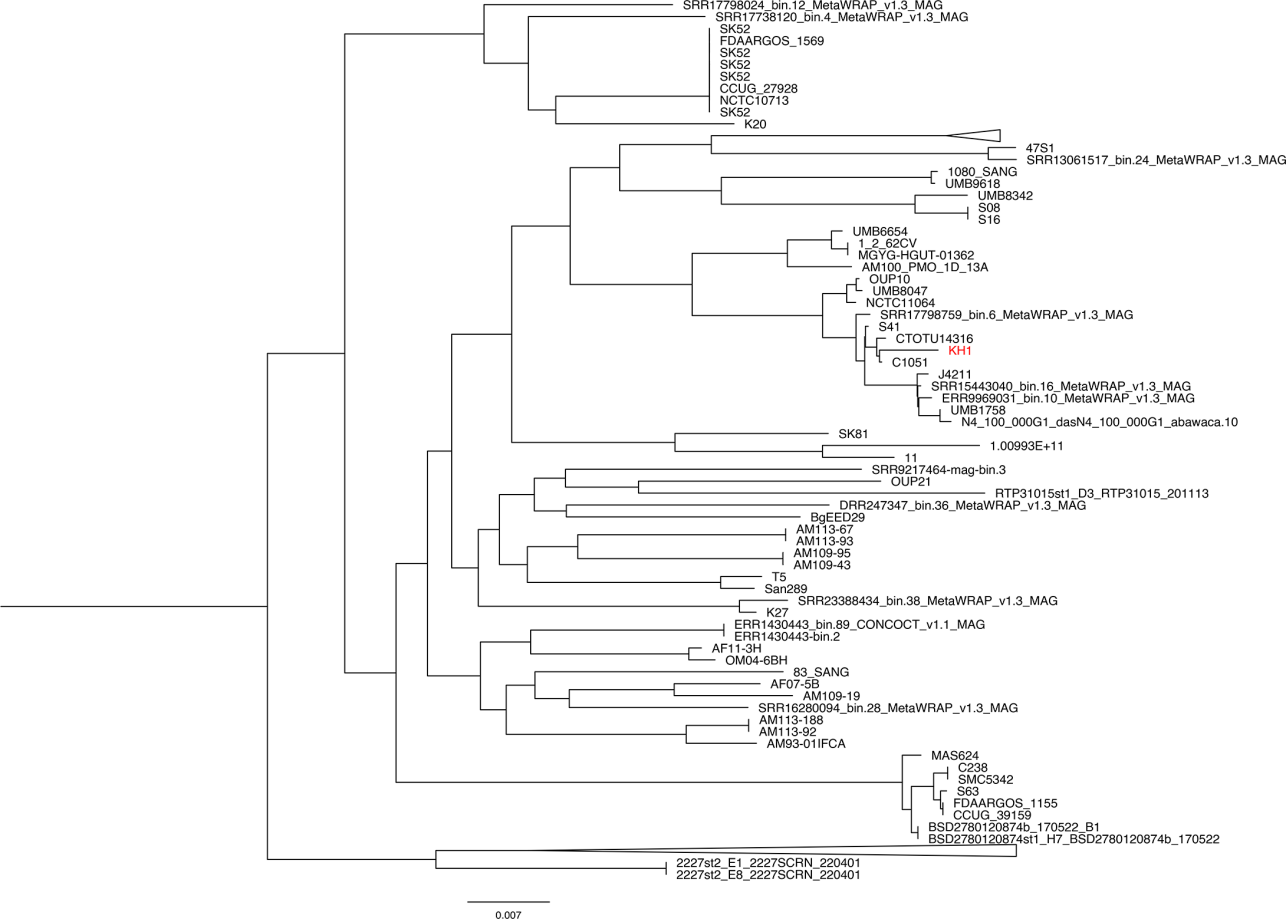
Phylogenetic analysis of *S. anginosus* KH1 and other *S. anginosus* strains. The tree was constructed from 355 *S. anginosus* genomes by iqtree2 (v2.1.2). Detailed information on the collapsed branches is listed in Fig. S1.

Upon analysing 355 accessible *S. anginosus* genomes, we found that the pan genome is comprised of 8316 genes, including 909 core genes (99%≤strains≤100%), 368 soft core genes (95%≤strains<99%), 1083 shell genes (15%≤strains<95%) and 5956 cloud genes (0%≤strains<15%). The large difference between the number of core and pan genomes indicates a significant variation among strains. Phylogenetic analysis unveiled three major clades, with one clade encompassing the majority of the strains, including KH1 (Figs 3 and S1). KH1 is phylogenetically closest to strain C1051 [51], which is also a blood isolate. According to a comparative genomic study by Prasad *et al.*, KH1 belongs to the group 1 genotype [52], which encompasses isolates from various human body sites, with the exception of urinary isolates, which is in agreement with the isolation origin of KH1.

Through pan genome analysis, eight unique genes were identified in KH1 (Table 3). Although the involvement of these unique genes in KH1 pathogenicity cannot be predicted based on annotation, it was noted that three unique genes exhibited a greater than threefold increase in expression when cultivated in the modified SM, compared to the artificial SV. Thus, it is tempting to suggest that variations at the genetic and expression levels among *S. anginosus* strains could affect the ability of the strain to transit from the oral cavity to the bloodstream, as demonstrated in *Escherichia coli* [53], and that the expression of unique genes may play a role in the survival of KH1 in the bloodstream.

**Table 3.** Unique loci on *S. anginosus* KH1 chromosome

Locus tag	Protein (aa)	Similarity*	Accession no.†	TPM		Fold-change
				SM‡	SV‡	SM/SV§
PO908_02850	129	Single-stranded DNA-binding protein of *Streptococcus* sp. HF-2466 (128 out of 129, 99%)	WP_195673100.1	48.5	15.6	3.1
PO908_02855	74	Hypothetical protein of *S. constellatus* (70 out of 71, 98%)	WP_314913874.1	41.6	8.5	4.9
PO908_02860	41	aa 1–39 of a hypothetical protein of *S. constellatus* (38 out of 39, 97%)	WP_314913875.1	33.8	7.8	4.3
PO908_03035	69	aa 824–889 of the phage tail tape measure protein of *Streptococcus* sp. HF-2466 (57 out of 66, 86%)	WP_195673119.1	0	0.3	0.0
PO908_03040	226	aa 922–1143 of the phage tail tape measure protein of *Streptococcus* sp. HF-2466 (216 out of 222, 97%)	WP_195673119.1	63.4	76.1	0.8
PO908_028225	532	Abi family protein of *Streptococcus gallolyticus* (531 out of 532, 99%)	WP_368395755.1	25.3	24.9	1.0
PO908_028230	635	ATP-binding protein of *Streptococcus sanguinis* (635 out of 635, 100%)	WP_002922332.1	37	30.9	1.2
PO908_028235	392	Hypothetical protein HMPREF9393_1502 of *S. sanguinis* SK1056 (391 out of 392, 99%)	EGJ37721.1	31.7	21.8	1.5

*Target with which the ORF shares the highest homology. The length of the alignment over the target size (number of aa in KH1/total number of aa in target) and the % similarity are listed in parentheses.

†Accession number of the locus listed in the similarity column.

‡SM, TPM from cells grown in modified SM; SV, TPM from cells grown in artificial SV.

§Fold increase in the modified SM (TPM in SM/TPM in SV).

### Genes encoding proteins involved in purine and glycogen biosynthesis, competence and putative bacteriocin production were induced during growth in modified SM

**Fig. 4.**
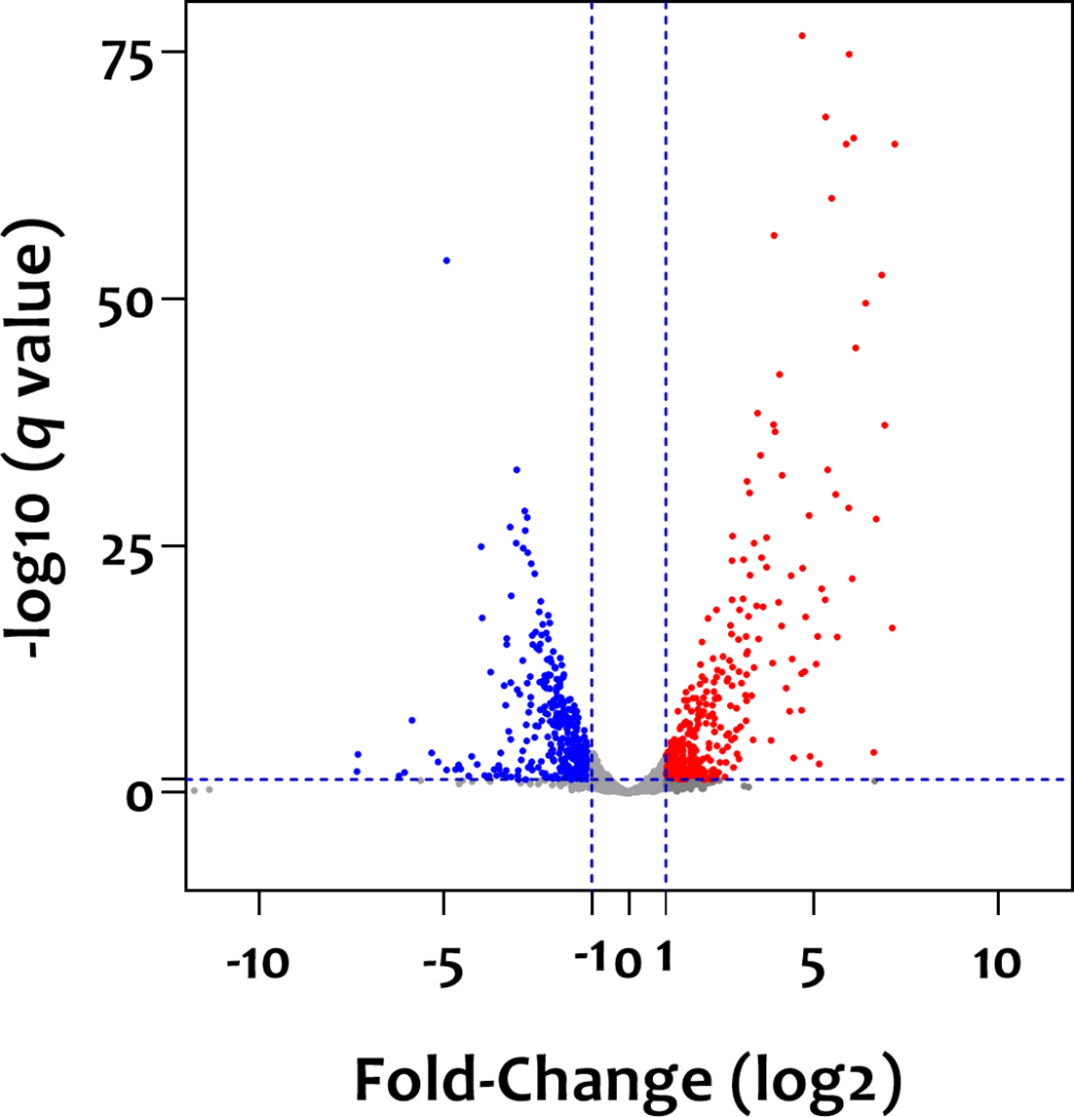
Volcano plot demonstrating the impact of culture medium on *S. anginosus* KH1 transcriptomes. Red and blue dots represent loci with a fold-change of ≥2 and ≤ 0.5 in modified SM compared to artificial SV, respectively. Loci with fold-changes between 0.5 and 2 or without significant expression changes are shown in grey.

As elevated gene expression in the modified SM, compared to expression levels in the artificial SV, may suggest the necessity of a particular gene for survival in the bloodstream, we sought to identify essential virulence genes by comparing transcriptomes of *S. anginosus* KH1 derived from growth in either of these conditions. Indeed, different transcriptomes were expressed by *S. anginosus* grown in these two types of media, reflecting altered gene expression in differing environments (Fig. 4). To be noted, loci exhibiting a TPM of less than ten were not included in the analysis as the low expression levels may not contribute significantly to the overall activity of KH1 under a specific growth condition. Three key findings resulted from the analysis of loci whose expression was elevated by ≥10-fold in the serum-like medium (Table 4). First, in agreement with an observation in *S. sanguinis* SK36 [54], most loci of the *pur* operon (PO908_00230–00300), responsible for purine biosynthesis, were significantly upregulated in the modified SM. Although the precise role of purine biosynthesis in pathogenesis remains undefined, studies in *Staphylococcus aureus* and *Enterococcus faecalis* indicated that induction of the *de novo* purine biosynthesis pathway is critical for the adaptation of these two species to growth in biofilms [55]. Additionally, a purine nucleotide transporter protein (TMPC) has been shown to act as a ligand for the adherence and invasion of gastric epithelial cells by *S. anginosus* ATCC33397 via TMPC-ANXA2 interaction [17]. This study further demonstrated that TMPC-ANXA2 interaction can activate the Mitogen-Activated Protein Kinase signalling pathway of the infected cells, leading to gastric inflammation, atrophy and even tumorigenesis in mice. Although we did not observe altered expression of the TMPC homologue in KH1 (PO908_05780) in our culture conditions, upregulation of purine biosynthesis suggests that this pathway plays a role in the survival of *S. anginosus* in hosts.

**Table 4.** Loci of *S. anginosus* KH1 upregulated ≥10-fold in modified SM

Locus tag	Gene*	Description	TPM		Fold-change
			SM†	SV†	SM/SV‡
**Metabolism**					
PO908_00245	*purM*	Phosphoribosylformylglycinamidine cyclo-ligase	204.9	19.6	10
PO908_00250	*purN*	Phosphoribosylglycinamide formyltransferase	195.1	19.5	10
PO908_00315	*dhaK*	PTS-dependent dihydroxyacetone kinase	457.2	30.2	15
PO908_01385	*rbsB*	Ribose import-binding protein	441	24.8	18
PO908_01400	–	Sugar-binding protein	321.9	28.7	11
PO908_03625	–	Dipeptidase	617.6	40.4	15
PO908_03940	*glgB*	1,4-*α*-Glucan branching enzyme GlgB	1384.5	34.7	40
PO908_03945	*glgC*	Glucose-1-phosphate adenylyltransferase	2248.9	36	62
PO908_03950	*glgD*	Glycogen biosynthesis protein GlgD	2043.8	34.5	59
PO908_03955	*glgA*	Glycogen synthase	1360.4	23.7	57
PO908_03960	*glgP*	Glycogen phosphorylase	951.9	21.3	45
PO908_06745	*gldA*	Glycerol dehydrogenase	1390.1	11.4	122
PO908_06750	*fsaA*	Fructose-6-phosphate aldolase	1129	10.9	104
PO908_06755	–	Formate C-acetyltransferase/glycerol dehydratase family glycyl radical enzyme	2152.9	14.7	146
PO908_06760	*licC_2*	Lichenan permease IIC	235.8	7.9	30
PO908_06765	*celA*	PTS cellobiose-specific IIB	440.2	8.9	49
PO908_06770	*licA_2*	Lichenan-specific PTS IIA	383.8	11.5	33
PO908_07955	*pdxK*	Pyridoxine kinase	1684.2	160.9	10
PO908_08475	*msmX*	Oligosaccharides import ATP-binding protein MsmX	431.3	44.2	10
PO908_09725	*malX*	Maltose maltodextrin-binding protein	724.1	75.1	10
PO908_10070	*tpiA_2*	Triosephosphate_isomerase	234.2	2	117
**Information storage and processing**					
PO908_01250	*comGA*	ComG operon protein	1367.2	30.3	45
PO908_01255	*comB*	Competence protein CglB	457.1	17.5	26
PO908_01260	*comGC*	Prepilin-type cleavage/methylation *N*-terminal domain protein	423.2	15.4	27
PO908_01265	*comGD*	Type II secretion system protein	768	18.4	42
PO908_01270	*comGE*	Type II secretory pathway, pseudopilin PulG	583.2	15.6	37
PO908_01275	*comGF*	Prepilin-type cleavage/methylation *N*-terminal domain protein	1435.9	20.4	70
PO908_01280	*comGG*	Competence protein	864.6	17.8	49
PO908_01380	*ssb_2*	Single-stranded DNA-binding protein	3270.2	24.3	135
PO908_01395	*lnrK*	Transcriptional regulatory protein LnrK	331.8	27.9	12
PO908_02290	*comFA*	ComF operon protein	262.1	7.4	35
PO908_02295	*comFC*	Putative competence protein FC	332.8	26.8	12
PO908_03730	–	Band-7–1 domain-containing protein	175.8	11.8	15
PO908_03735	–	DNA-directed RNA polymerase subunit P	319.8	15.2	21
PO908_05005	*ComEA*	ComEA family DNA-binding protein	756.6	12.2	62
PO908_05010	*comEC*	ComE operon protein	345.2	10	35
PO908_05500	*dprA*	SAM-DrpA domain-containing protein	620	9.4	66
**Cellular processes and signalling**					
PO908_00330	*glpF*	Aquaporin	335.8	25.3	13
PO908_03520	*lcnC*	Lactococcin-A transport/processing ATP-binding protein LcnC	166.4	14.6	11
PO908_03560	*blp3.6*	Putative Blp family class II bacteriocin	430.8	10.9	40
PO908_03570	*blp3.4*	Putative bacteriocin Blp3.4	255.4	10	26
PO908_03575	*blp3.3*	Bacteriocin immunity protein	121.9	8.4	15
PO908_03580	*blp3.2*	Hypothetical protein	299.7	11.1	27
PO908_03585	*blp3.1*	Putative Blp family class II bacteriocin	255.8	10	26
PO908_10065	*galE_2*	UDP-glucose 4-epimerase	624.3	4	156
**Uncharacterized**					
PO908_00320	*dhaL*	PTS-dependent dihydroxyacetone kinase, ADP-binding subunit DhaL	34.5	581.5	17
PO908_00325	*dhaM*	PTS-dependent dihydroxyacetone kinase, phosphotransferase subunit DhaM	26.1	344.6	13
PO908_01790	–	Hypothetical protein	1155.5	17	68
PO908_03515	–	LAL-C2 domain-containing protein	237	13.5	18
PO908_03530	–	Hypothetical protein	244.5	11.4	21
PO908_03590	–	Putative bacteriocin transport accessory protein	182.2	8.3	22
PO908_03595	–	Hypothetical protein	486	16.6	29
PO908_03740	–	TPM domain-containing protein	225.3	13.6	17
PO908_05015	–	Hypothetical protein	185.9	9.2	20
PO908_06190	–	UPF0758 protein SpyM3_0777	191.6	10.1	19
PO908_07950	*hmpT*	Integral membrane protein	1418.1	91.6	15
PO908_08610	–	Hypothetical protein	432.3	35.8	12
PO908_08620	–	HAD superfamily hydrolase	213.9	19.5	11
PO908_09650	*cinA*	Putative competence-damage inducible protein	1815.5	70.5	26
PO908_10075	*mdaB*	Putative flavin reductase	32.6	0.2	163
PO908_10110	–	Hypothetical protein	2202.1	25.9	85

*The proposed gene name.

†SM, TPM from cells grown in modified SM; SV, TPM from cells grown in artificial SV.

‡Fold increase in the modified SM (TPM in SM/TPM in SV).

Second, though the modified SM is nitrogen-rich, it was surprising to find that genes involved in glycogen biosynthesis (PO908_03940–03960) were upregulated by ~40- to 60-fold. Nonetheless, a study [56] in *Shigella flexneri* found that intracellular *S. flexneri* expresses a higher level of glycogen branching enzyme (GlgB) and glucose-1-phosphate adenylyltransferase (GlgC), compared to its extracellular counterpart, suggesting that glycogen production under glucose-limiting conditions could optimize the fitness of intracellular *S. flexneri*. Thus, it is possible that the upregulation of glycogen production in the modified SM is not for carbon nutrient storage, but rather that *S. anginosus* employs glycogen biosynthesis to facilitate its transition from the oral cavity to the bloodstream via an unknown mechanism.

Third, genes associated with both competence development (PO908_01250–01280, 02290–02295 and 05005–05010) and bacteriocin production (PO908_03560–03595) were upregulated in the modified SM, suggesting that these two systems are sensitive to the same environmental cues. The coordinate regulation of competence and bacteriocin production has also been described in *Streptococcus pneumoniae* [57], in which the competence peptide could induce the expression of pneumocin. It is proposed that such regulation allows *S. pneumoniae* to gain additional genotypes, such as antibiotic resistance, from competing bacteria. Thus, it is possible that upregulation of bacteriocin production and acquisition of extracellular DNA could optimize the fitness of *S. anginosus* in the bloodstream.

### Expression of loci involved in quorum quenching and translation was elevated in the artificial SV

A total of 377 loci exhibited higher expression when grown in the artificial SV than the modified SM, though the level of increase was less pronounced compared to upregulation in the modified SM. Specifically, 13 loci showed a ≥10-fold increase in expression in the artificial SV (Table 5), compared to 61 loci in the modified SM (Table 4). Three key observations were noted. First, the expression of a putative quorum-quenching synthesis system, including a LysR family regulator (PO908_05535), an acyl-CoA dehydrogenase (PO908_05530) and a putative lactonase YtnP (PO908_05525), was upregulated more than tenfold. Lactonase YtnP is able to interrupt bacterial signalling based on acyl homoserine lactones and has been identified in both Gram-positive and Gram-negative bacteria. Specifically, YtnP of *Bacillus paralicheniformis* ZP1 inhibits biofilm formation and breaks down mature *P. aeruginosa* PAO1 biofilms [58]. As the putative YtnP is a cytoplasmic protein, it is possible that the release of lactonase from lysed *S. anginosus* could optimize the competitiveness of the remaining * S. anginosus* population in the oral niche by interrupting the quorum sensing regulation of other bacteria in the same niche.

**Table 5.** Loci of *S. anginosus* KH1 downregulated ≥10-fold in modified SM

Locus tag	Gene*	Description	TPM		Fold-change
			SM†	SV†	SM/SV‡
**Metabolism**					
PO908_05530	*acdA*	Acyl-CoA dehydrogenase	18.7	193.4	0.10
PO908_05555	*thlA*	Acetyl CoA acetyltransferase	8.9	87.6	0.10
PO908_05560	–	3-Hydroxybutyryl-CoA dehydrogenase	9.1	145	0.06
PO908_07025	*lacB*	Galactose 6 phosphate isomerase subunit	0.9	11.9	0.08
PO908_07040	*cshA*	DEAD box ATP-dependent RNA helicase CshA	18.4	561.5	0.03
PO908_08785§	*pox5*	Pyruvate oxidase	41.3	341.5	0.12
**Cellular processes and signalling**					
PO908_00045	–	Putative cell division protein DivIC	8.9	84.5	0.11
PO908_07195	–	FtsX-like permease family protein	2	27.3	0.07
**Information storage and processing**					
PO908_00040	–	RNA-binding S4 domain-containing protein	1	17.3	0.06
PO908_00775	*xerC_2*	Tyrosine recombinase XerC	NE	17.4	na
PO908_05535	–	LysR family transcriptional regulator	4.8	75.7	0.06
PO908_06815	*thrS*	Threonine tRNA ligase	63.9	631.6	0.10
**Poorly characterized**					
PO908_00020	*yehR*	Putative lipoprotein YehR	4.6	50.5	0.09
PO908_00780	–	Hypothetical protein	NE	22.8	na
PO908_00840	–	Hypothetical protein	NE	19.2	na
PO908_05525	*ytnP*	Putative quorum-quenching lactonase YtnP	8.2	82.8	0.10
PO908_06020	–	Hypothetical protein	2	22.8	0.09
PO908_09935	*smc_5*	Chromosome partition protein Smc	7.9	465.5	0.02

*The proposed gene name.

†SM, TPM from cells grown in modified SM; SV, TPM from cells grown in artificial SV; NE, not expressed.

‡Fold-increase in the modified SM (TPM in SM/TPM in SV); na, not applicable.

§PO908_08785 exhibits a 8-fold downregulation in modified SM.

**Fig. 5.**
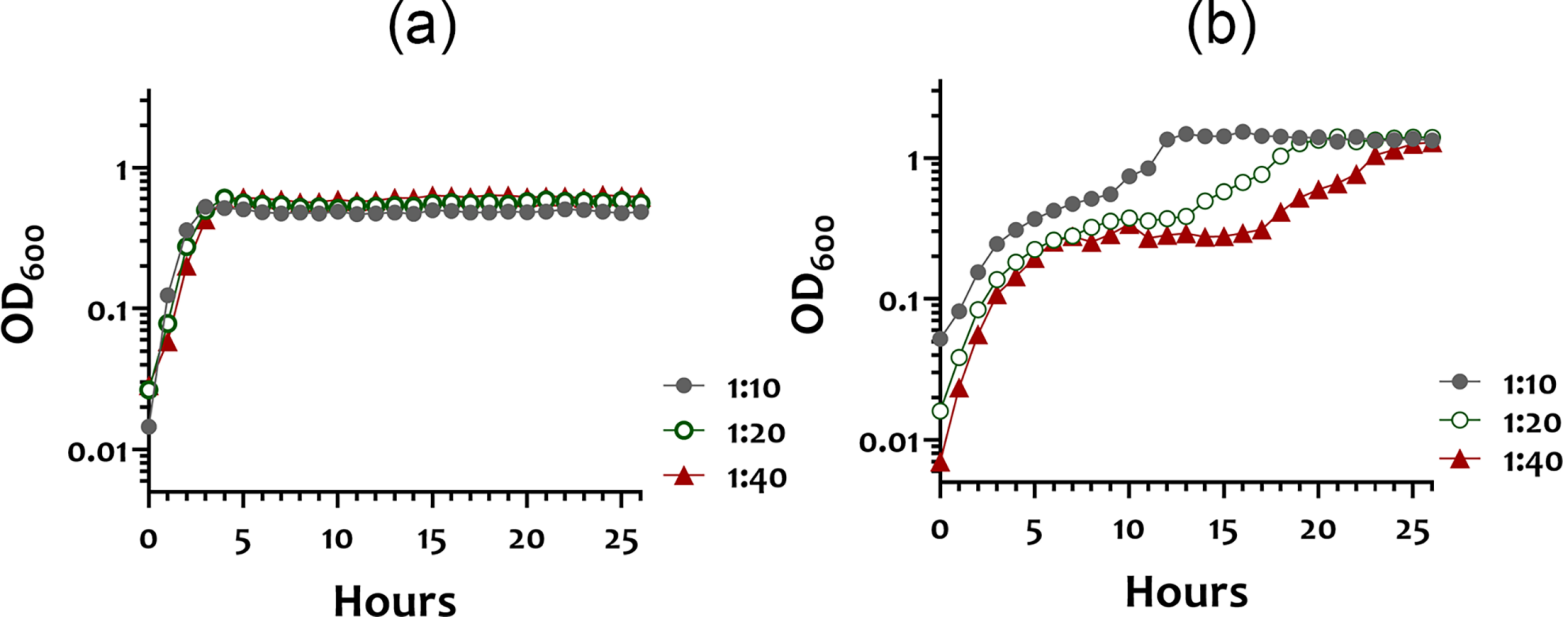
The growth of *S. anginosus* KH1 in artificial SV (**a**) and modified SM (**b**). Overnight cultures of KH1 grown in TH medium were diluted into the respective medium at 1 : 10, 1 : 20 or 1 : 40. The presented result is the representative of three biological replicates.

Second, genes encoding several enzymes involved in ribosome biosynthesis, translation and RNA decay (PO908_00040, 06815 and 07040) and cell division (PO908_00045 and 07195) showed higher expression in the artificial SV compared to the modified SM. In contrast, transcription of these groups of genes was not affected by growth in the modified SM, suggesting that *S. anginosus* adapted to the artificial SV more efficiently. Indeed, when a culture of *S. anginosus* KH1 grown in TH broth overnight was subcultured into artificial SV, we observed comparable growth kinetics to a culture grown in TH, with a generation time of 45 min and a final yield at OD_600_≈0.6, in all inoculum dilutions tested (Fig. 5a). In contrast, KH1 exhibited a diauxic growth pattern when subcultured into the modified SM, where the length of the transition period was more evident at a lower inoculum size (Fig. 5b). KH1 exhibited a generation time of 2 h and a final OD_600_ of 0.1 in the first growth phase, but the final yield achieved an OD_600_=1 in all dilutions tested, suggesting that KH1 is equipped to persist in the bloodstream.

Finally, the expression of pyruvate oxidase (PO908_08785) in artificial SV, an enzyme catalysing H_2_O_2_ production, was eightfold higher than that in the modified SM, suggesting that H_2_O_2_ production could provide a competitive advantage for *S. anginosus* in the oral cavity, as has been reported previously in *S. sanguinis*, which can antagonize *Streptococcus mutans* by inflicting oxidative damage via reactive oxygen species [59].

## Conclusions

The genome of *S. anginosus* KH1 is shaped by chromosomal duplication and HGT events, and the large size of the pan genome reflects the variations among *S. anginosus* strains. The environmentally dependent differential expression of specific loci examined in this study strongly suggests that *S. anginosus* KH1 is equipped to maintain a niche in the oral cavity and to survive in the bloodstream. The genome characterization and transcriptomic analysis of *S. anginosus* KH1 described here provide insights into the physiology and pathogenic potential of this bacterium.

## Supplementary material

10.1099/mic.0.001535 Uncited Fig. S1.

10.1099/mic.0.001535 Uncited Supplementary Material 1.

10.1099/mic.0.001535 Uncited Supplementary Material 2.

## Abbreviations

CARD: comprehensive antibiotic resistance database
CF: cystic fibrosis
COG: Clusters of Orthologous Groups
Em: erythromycin
HGT: horizontal gene transfer
MBC: minimal bactericidal concentration
SAG: *Streptococcus anginosus* group
SM: serum medium
SV: saliva medium
TH: Todd Hewitt
TPM: transcripts per million
