# Supplementary figures and images for: Genomic and transcriptomic insights into the virulence and adaptation of shock syndrome-causing Streptococcus anginosus
Source: Microbiology (Reading). 2025 Feb 20;171(2):001535. doi: 10.1099/mic.0.001535 (PMC12282319; doi:10.1099/mic.0.001535)

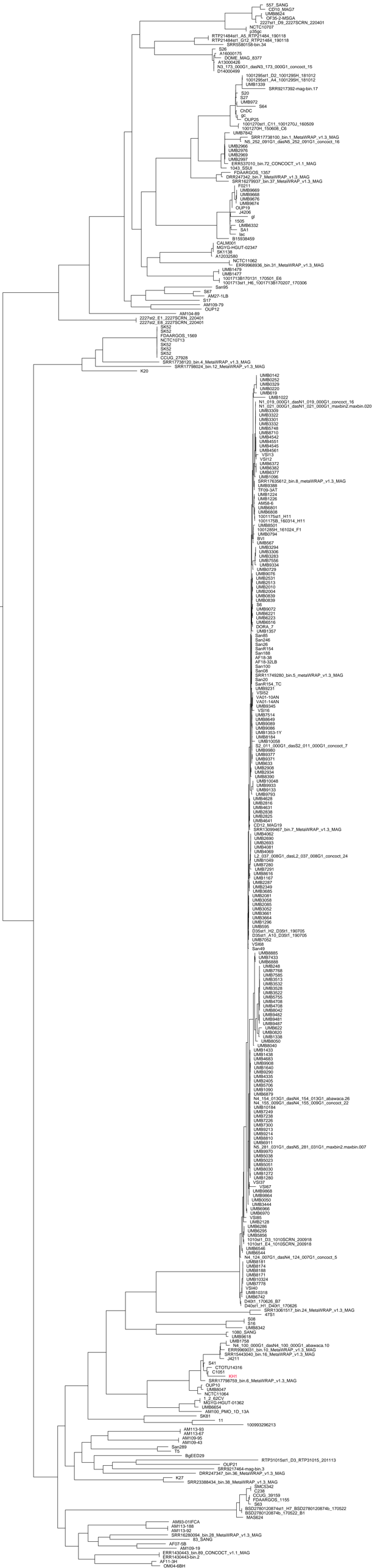

Supplement: Uncited Fig. S1. [file mic-171-01535-s001.pdf]
